# The abundance of *cis*-acting loci leading to differential allele expression in F1 mice and their relationship to loci harboring genes affecting complex traits

**DOI:** 10.1186/s12864-016-2922-9

**Published:** 2016-08-11

**Authors:** Seungeun Yeo, Colin A. Hodgkinson, Zhifeng Zhou, Jeesun Jung, Ming Leung, Qiaoping Yuan, David Goldman

**Affiliations:** 1 Laboratory of Neurogenetics, National institute on Alcohol Abuse and Alcoholism, National Institutes of Health, Bethesda, MD 20852 USA; 2 Laboratory of Epidemiology and Biometry, National institute on Alcohol Abuse and Alcoholism, National Institutes of Health, Bethesda, MD 20852 USA

**Keywords:** RNA-Seq, Differential allelic expression, *cis*-acting loci, Quantitative trait loci

## Abstract

**Background:**

Genome-wide surveys have detected *cis*-acting quantitative trait loci altering levels of RNA transcripts (RNA-eQTLs) by associating SNV alleles to transcript levels. However, the sensitivity and specificity of detection of *cis*- expression quantitative trait loci (eQTLs) by genetic approaches, reliant as it is on measurements of transcript levels in recombinant inbred strains or offspring from arranged crosses, is unknown, as is their relationship to QTL’s for complex phenotypes.

**Results:**

We used transcriptome-wide differential allele expression (DAE) to detect cis-eQTLs in forebrain and kidney from reciprocal crosses between three mouse inbred strains, 129S1/SvlmJ, DBA/2J, and CAST/EiJ and C57BL/6 J. Two of these crosses were previously characterized for *cis*-eQTLs and QTLs for various complex phenotypes by genetic analysis of recombinant inbred (RI) strains. 5.4 %, 1.9 % and 1.5 % of genes assayed in forebrain of B6/129SF1, B6/DBAF1, and B6/CASTF1 mice, respectively, showed differential allelic expression, indicative of *cis*-acting alleles at these genes. Moreover, the majority of DAE QTLs were observed to be tissue-specific with only a small fraction showing *cis*-effects in both tissues. Comparing DAE QTLs in F1 mice to *cis*-eQTLs previously mapped in RI strains we observed that many of the cis-eQTLs were not confirmed by DAE. Additionally several novel DAE-QTLs not identified as cis-eQTLs were identified suggesting that there are differences in sensitivity and specificity for QTL detection between the two methodologies. Strain specific DAE QTLs in B6/DBAF1 mice were located in excess at candidate genes for alcohol use disorders, seizures, and angiogenesis previously implicated by genetic linkage in C57BL/6J × DBA/2JF2 mice or BXD RI strains.

**Conclusions:**

Via a survey for differential allele expression in F1 mice, a substantial proportion of genes were found to have alleles altering expression in *cis*-acting fashion. Comparing forebrain and kidney, many or most of these alleles were tissue-specific in action. The identification of strain specific DAE QTLs, can assist in assessment of candidate genes located within the large intervals associated with trait QTLs.

**Electronic supplementary material:**

The online version of this article (doi:10.1186/s12864-016-2922-9) contains supplementary material, which is available to authorized users.

## Background

Gene expression is regulated by multi-layered genetic and epigenetic mechanisms. Genetic variation can lead to inter-individual variation in gene expression through both *cis*- effects at genes and by *trans*-acting mechanisms. *Trans*-acting factors may affect transcription of target genes irrespective of genomic location, or modulate levels of transcripts derived from genes located on either the paternally or maternally derived chromosome. *Trans*-acting factors most obviously include DNA-binding transcription factors [1], but also any of the other diverse molecular actors, microRNA’s, ribosomal proteins, rRNA’s and ions, that can alter transcription, or RNA processing, or RNA stability, or RNA translation. C*is*-regulatory elements modulating transcript expression are usually located nearby or within genes, but at the minimum are on the same chromosome. Genetic variations in *cis*-regulatory elements located anywhere upstream, downstream, within introns, and as well as in the 5’ and 3’ un-translated regions of genes, and even at considerable distances from genes, can alter transcription, mRNA stability, mRNA processing efficiency, or mRNA isoform expression [2]. The importance of genomic elements that regulate gene expression was highlighted in a series of studies arising from the ENCODE project, suggesting that a significant proportion of the human genome might regulate gene expression [3].

Genetic variation altering gene expression plays a critical role in health and disease. For example, regulatory variants located 14 kb upstream and 8 kb downstream from the lactase gene lead to persistence of expression of the lactase gene in adulthood, and lactose intolerance [4]. In behavioral genetics, a polymorphism in the immediate 5’ upstream region of the neuropeptide Y gene (*NPY*) alters transcription of this gene and, through the role of this neuropeptide in stress response and emotion, alters susceptibility to stress and pain [5]. Variants affecting transcript levels may be particularly important in neurogenic disorders [6, 7] including schizophrenia for which some 108 genes were recently implicated by genome wide association [8], but only a few functional loci have actually been identified [9]. Genome-wide association studies of other complex disorders have implicated many chromosomal regions. However, the causal functional genetic variants, many of which appear to be located far from any known gene, remain largely unknown.

Despite the importance of identifying *cis*-acting functional loci that alter gene expression, progress has been limited and achieved largely on a gene-by-gene basis. Genetic methods associating SNP and SNV (single nucleotide variant) alleles to RNA levels measured by microarray or RNA-Seq have been used to identify *cis* and *trans*-eQTLs, but this approach only infers causality for any particular variant by association [10]. Although a large number of putative *cis*- and *trans*- expression quantitative trait loci (eQTLs) have been located in human and rodents by genetic methods, most have not been validated at the level of molecular function.

Differential allele expression (DAE) analysis by RNA-Seq is a genomic molecular approach that can overcome many of the main biological limitations in detection of *cis*-acting loci. DAE requires the presence of a heterozygous reporter variant in the transcript. DAE QTLs are in this regard *cis*-eQTLs. DAE can confirm *cis*-acting eQTLs identified by genetic approaches but can also be an exquisitely sensitive and potentially more accurate tool for detection. By measuring the differential expression of heterozygous reporter alleles within the same cell, tissue and individual, the differential actions of *cis* alleles in proximity to the reporter locus are observed in the same context. The relative expression of transcribed alleles at heterozygous reporter loci is measured. Using reciprocal crosses, DAE can also detect parental imprinting of genes due to epigenetic events including DNA methylation, histone modification, non-coding RNAs (ncRNAs) and chromatin organization that are mechanistic in the mono-allelic expression of autosomal genes [6]. DAE has previously been used in F1 mice to identify strain -specific DAE QTLs and effects of genomic imprinting. Several studies have reported allelic imbalance in F1 mice using RNA-Seq [11–19]. Most of these studies have focused on parent-of-origin effects, as seen for imprinted genes [13–16, 18, 19]. Inconsistencies that have been observed in the studies arose in part because of technical differences [13, 16]. However, two types of parental imprinting patterns have been reported via DAE: a classical imprinted pattern in which there is full allelic silencing and allelic imbalance [11–13, 16, 19].

Reports using DAE to detect strain-specific DAE QTLs have been limited. Analysis of expression in liver of reciprocal F1 crosses between C57BL/6J and CAST/EiJ [12] and crosses between C57BL/6J and DBA/2J [17] found that approximately 14 % of assayable genes, 1,391/10,090 [12] and 284/2256 [17], had RNA *cis*-eQTLs in both crosses even though the number of assayable genes, which is to say expressed genes with SNVs in transcripts, varied between the two studies. RNA-Seq DAE performed on brain from three different reciprocal F1 mouse crosses including the wild derived inbred strains, CAST/EiJ, PWK/PhJ, and WSB/EiJ suggested that greater than 80 % of assayable genes have DAE QTLs, with approximately 64 % of these being detectable in any one of the three reciprocal crosses [11]. This latter study suggested that the great majority of genes have a *cis*-acting functional locus modulating RNA expression, but the findings between studies are clearly discrepant, and an intent of our study was to distinguish between these two highly disparate estimates of the abundance of *cis*-acting loci.

We used RNA-Seq to identify DAE QTLs that may alter behavior or other genetically influenced phenotypes. We analyzed transcriptomes of two tissues (forebrain and kidney) of F1 mice derived from reciprocal crosses between the C57BL/6J (B6) inbred mouse strain and strains with which it is known to display distinct behavioral characteristics, 129S1/SvlmJ (129S) and DBA/2J (DBA), as well as the more genetically divergent wild-derived inbred strain, CAST/EiJ (CAST). DAE is assayable only for genes whose transcripts contain SNVs that differentiate the two parental strains. In the absence of *cis*-acting alleles or imprinted elements, genes transmitted from either parent will be equally expressed. DAE QTLs were validated by direct Sanger sequencing of RT-PCR products, by RT-qPCR quantitation of mRNA levels in parental strains, and by the ability of DAE to reliably detect known imprinted loci and X chromosome-encoded genes, in the latter male F1 mice. Using RNA-Seq DAE we estimated the fraction of transcripts assayable for *cis*-eQTLs within these crosses and the number of *cis*-eQTLs active in forebrain and kidney. We also compared the sensitivity and specificity of DAE analysis for the identification of *cis*-acting loci modifying transcript levels to eQTLs identified in the relevant mouse strains and explored the relevance of DAE QTLs to genes implicated in complex traits by genetic linkage analyses in these strains.

## Results

### Genome-wide identification of DAE QTLs in forebrain and kidney of F1 mice by RNA-Seq

Reciprocal crosses were made between C57BL/6J (B6) and three other inbred strains: 129S1/SvlmJ (129S), DBA/2J (DBA), and CAST/EiJ (CAST). PolyA^+^ mRNA was prepared from forebrain and kidney of F1 mice generated from these crosses and mRNA was pooled from six individual F1 mice for each transcriptome library. In the twelve sequencing analyses between 17.8 and 45.4 million sequence fragments, corresponding to 0.64–1.75 Gb, were mapped (Additional file 1: Table S1). Mapped reads were analyzed for sequence variation in order to determine whether either parental allele was preferentially expressed. When the B6 reference sequence (NCBI37/mm9) was used to map F1 RNA-Seq reads, allelic mapping bias was detected, evidenced by the appearance of an excess of B6 allele specific reads in both sides of any reciprocal cross. We also observed the existence of reference bias by mapping sequence reads to a reference in which other strain specific variants were introduced. Reference bias was eliminated by mapping data to both parental reference genomes and averaging results; the equivalent of mapping to a haplotypically accurate heterozygous reference sequence (Additional file 2: Figure S1). Strain-specific alleles were validated through exome sequencing of the parental mouse strains and by comparison to the Sanger mouse genome resource (The VCF file 20111102-snps-all.annotated.vcf.gz was downloaded from ftp://ftp-mouse.sanger.ac.uk/REL-1105) to ensure that only genuine SNVs were interrogated (Additional file 1: Table S2).

DAE was analyzed in forebrain and kidney for the three reciprocal crosses. Only SNVs with read counts ≥8 were included, this being the threshold at which DAE was consistently detected for known maternal and paternally imprinted genes (Additional file 1: Table S3). Differentially allelic expressed transcripts were observed in both tissues for all three reciprocal crosses (Additional file 2: Figure S2). In each instance there was a similar ratio of over-represented strain-specific alleles in all analyses, indicating that reference bias was successfully eliminated. The number of genes assayed for DAE varied depending upon the number of strain-specific SNVs detectable and the tissue. Using these criteria, approximately 3,000–5000 genes were assayable for DAE in forebrain and 2,500 – 4,500 genes in kidney. Using a FDR cutoff of *p* ≤ 0.05 and requiring the same direction of expression in each F1 cross, 3.2 – 9.7 % of assayed genes in forebrain showed significant allelic imbalance in expression. Although kidney had fewer assayable genes for DAE analysis than forebrain, it had higher proportion of genes with evidence of DAE QTLs (7.6–19.5 %) (Additional file 2: Figure S2).

**Fig. 1:**
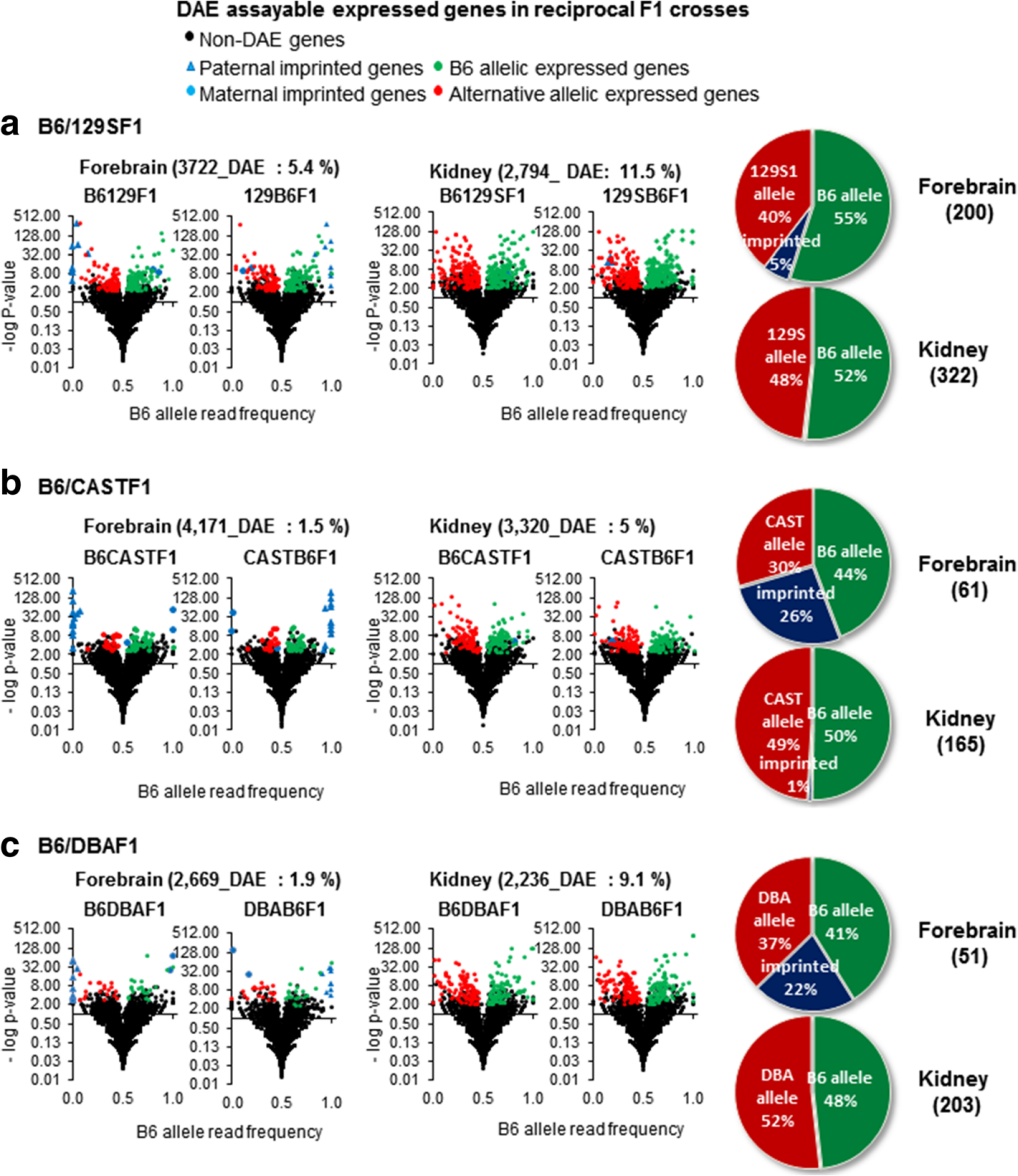
Distribution of DAE genes with strain specific imbalance and imprinted genes in forebrain and kidney. Volcano plots depict B6 allele read frequencies (0–1) against FDR corrected (−log_10_) P values for significance of allelic imbalance for (**a**) B6/129SF1, (**b**) B6/CASTF1, and (**c**) B6/DBAF1 reciprocal crosses in forebrain and kidney. Pie charts depict relative frequencies of over represented parental alleles and imprinted genes. The number of genes with *cis*-loci altering transcript expression as detected by RNA-Seq DAE (DAE QTLs) observed in both reciprocal crosses is shown below the tissue, with the cross in (parentheses)

Identification of DAE QTLs is enhanced by observation of consistency of DAE in reciprocal F1 crosses. When DAE effects are not consistent in reciprocal crosses, this can identify genes imprinted according to parental origin. Using DAE patterns we were able to identify imprinted genes and distinguish these from strain-specific DAE QTLs. The numbers of genes exhibiting DAE varied between the different F1 crosses, and between tissues (Fig. 1): 5.4 % (200/3,722), 1.9 % (51/2,669) and 1.5 % (61/4,171) of expressed genes showed DAE in forebrain in B6/129SF1(B6129SF1 and 129SB6F1), B6/DBAF1(B6DBAF1 and DBAB6F1), and B6/CASTF1(B6CASTF1 and CASTB6F1) reciprocal crosses respectively, and for 11.5 % (322/2,794), 9.1 % (203/2,236) and 5.0 % (165/3,320) of expressed genes in kidney in the B6/129SF1, B6/DBAF1, and B6/CASTF1 reciprocal crosses respectively.

The overlap in genes showing significant DAE in both reciprocal crosses was greater than expected by chance (Additional file 1: Table S4). Surprisingly, after FDR correction the number of DAE QTLs was smallest in B6/CAST F1 mice, despite the fact that there are a larger number of informative SNVs in this cross than for the other crosses. This observation is possibly explained in a comparison of the forebrain data from B6/CASTF1 with B6/129SF1 which showed that most of the potentially informative SNVs for the B6/CAST F1 mice were located in transcripts with low sequence read number even though the B6/CAST F1 mice had higher mapped read counts than the other crosses (Additional file 1: Table S1). Analysis of DAE using a preselected set of transcribed regions (or “genes”) could potentially implicate additional B6/CAST loci as having DAE QTLs. However, our pipeline was designed to test DAE in transcripts regardless of whether the transcript was associated with an annotated gene, and in keeping with the potential, and at times already demonstrated, functional significance of some non-coding RNAs.

### Validation for strain-specific and parental allele specific DAE-QTLs in the forebrain

We tested the ability of our RNA-Seq based pipeline to reliably detect DAE QTLs in multiple ways. Because analyses were performed on male F1 mice, only the maternal allele should be detectable in transcripts derived from the X chromosome, and this was confirmed for almost all the DAE assayable X-linked genes in all the F1 crosses (Additional file 2: Figure S3). Next, DAE results for 20 randomly selected genes with SNVs showing significant DAE in forebrain (FDR *p*-value ≤ 0.05) and 15 genes with SNVs showing no DAE, were validated in the individual F1 mice originally pooled for RNA-Seq by quantitative Sanger sequencing using cDNA. Sequence from genomic DNA of the same F1 mice was used to control and correct for any bias in allelic quantitation. Findings from the quantitative Sanger sequencing showed excellent correlation with RNA-Seq allelic expression imbalance (or the lack thereof) for all 35 genes analyzed (Additional file 2: Figure S4B). The validity of RNA-Seq to identify genuine DAE QTLs was further demonstrated by the detection of multiple previously identified imprinted genes in forebrain [13, 20–23]. These included 16 imprinted genes (*Zrsr1*, *Nap1l5*, *Calm1*, *Snrpn*, *Peg3*, *Ndn*, *Dlk1*, *H13*, *Zdbf2*, *Sgce*, *Peg10*, *Usp29*, *Inpp5f*, *Rasgrf1*, *Copg2* and *Impact*) and 7 imprinted non-coding RNAs (*Apeg3*, *Rian*, *Snord64*, *Meg3*, *D7Ertd715e*, *Peg13* and *C230091D08Rik*). DAE of several of these imprinted genes and non-coding RNAs was confirmed by quantitative Sanger sequencing (Additional file 2: Figure S5). We uncovered at least one instance of a gene, *Zim3*, that has been reported as maternally imprinted in mouse testis [20] but which shows strong DAE in forebrain where only the 129S- derived transcript is expressed in either reciprocal F1. Further, in the parental strains, 129S mice express *Zim3* at levels 6-fold higher than B6 mice (Additional file 2: Figure S8). The DAE in reciprocal F1 mice and the differing levels of *Zim3* expression in the parental strain together suggest that, at least in forebrain, *Zim3* expression is not regulated by imprinting, but by *cis*-acting functional elements that vary between B6 and 129S mice. Direct Sanger sequencing of *Zim3* cDNA from the F1 animals also indicated the presence of two alternatively spliced transcripts, however individual F1 mice only expressed one of these two isoforms, the expressed transcript always being derived from the 129S allele suggesting a complex regulation of this locus.

For comparison to the genetic approach for detecting eQTLs of measuring RNA levels in RI strains and to further confirm DAE QTLs, we measured transcript levels in parental strains for 10 genes that exhibited DAE QTLs in the F1 mice. Expression levels were normalized to the expression in B6 mice and the ratios of expression levels compared to the RNA-Seq and quantitative Sanger sequencing allelic imbalance ratios observed in the F1 mice. There was good correlation between the relative parental expression levels and the ratio of allelic expression observed in the F1 mice. These multi-level results, including consistent allelic imbalance in the two reciprocal F1 strains identified by RNA-Seq, replication of the allelic ratios by quantitative Sanger sequencing, and the coherent differential gene expression in the parental strains not only confirm strain-specific DAE QTLs, but also demonstrate the value of using RNA-Seq for genome-wide identification of *cis*-eQTLs altering gene transcript levels (Additional file 2: Figure S4C).

### Tissue-specific and shared DAE QTLs between forebrain and kidney

The ratios of genes in which either the B6 allele or alternative allele was preferentially expressed was similar in all three reciprocal crosses, for both forebrain and kidney. DAE QTLs can be identified based on consistency of DAE in the reciprocal crosses, by extent of differential expression, and by level of statistical significance, or by a combination of these parameters. We have in effect done this by pruning the genes analyzed for DAE by level of representation in the sequencing, prior to applying statistical testing, and by use of reciprocal crosses.

**Fig. 2:**
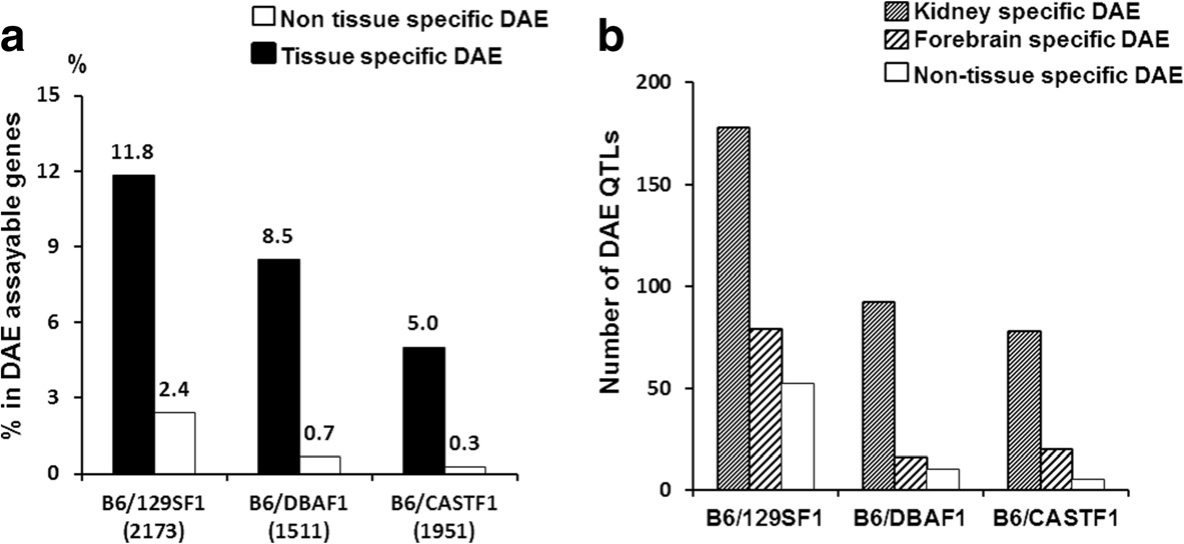
Tissue specific and non-tissue specific DAE QTLs in forebrain and kidney. **a** Tissue specific and non-tissue specific DAE QTLs. The X-axis indicates the number of co-expressed DAE-assayable genes in forebrain and kidney of each reciprocal F1 cross. Y-axis is the ratio of tissue specific DAE QTL genes and non-tissue specific DAE QTL genes in all genes that are expressed in both forebrain and kidney, and that have SNVs, making them assayable for DAE in both tissues. **b** Kidney, forebrain, and non-tissue specific DAE QTLs. Y axis indicates number of DAE QTLs

Tissue-specific DAE was common, but in line with previous lower estimates. Only a small proportion of genes showed conservation of DAE pattern between forebrain and kidney even though the same combinations of DNA control elements will be present to regulate gene expression across different forebrain and kidney samples. Tissue-specific cellular function is reflected in part by different patterns of gene expression between tissue types. Analysis of expressed genes with SNVs detected in both forebrain and kidney in both reciprocal crossed F1 mice identified 2,173 genes in B6/129SF1, 1,511 genes in B6/DBAF1, and 1,951 genes in B6/CASTF1. Within this subset of genes 11.9 %, 8.5 %, and 5.0 % showed forebrain DAE QTLs in B6/129S F1, B6/DBA F1 and B6/CAST F1, respectively, but only a small proportion of DAE genes showed DAE in both forebrain and kidney: 2.4 % in B6/129SF1, 0.7 % in B6/DBAF1, and 0.3 % in B6/CASTF1 (Fig. 2a). Also, we observed that for the subset of genes expressed in both kidney and forebrain that more genes showed DAE in kidney than in forebrain (Fig. 2b). Moreover, in forebrain 3 genes, *Acadm*, *Aldh7a1*, and *Dci*, showing preferential BL6 allelic expression showed 129S-allelic preference in kidney in both F1 crosses, suggesting that *cis*-acting loci at these genes were regulated by tissue specific trans-factors. These tissue-specific DAE findings are unlikely to be significantly confounded due to variation in detection power, which is affected by both tissue-specific expression level and sequence coverage, because our comparison only included genes detected at ≥ 8 reads in both tissues, and which showed similar allelic preference for a tissue in both reciprocal crosses. Additionally no bias was observed towards tissue where more sequencing read counts are obtained as would be expected if expression level was influencing the results. These data show that the majority of co-expressed genes in both kidney and forebrain were expressed with a preference for a specific allele in only a single tissue type illustrating the importance of tissue-specific factors in transcriptional regulation, and RNA processing and stability.

### Important genes in neural functions and behavior show strain-specific differential allelic patterns in the forebrain

**Fig. 3:**
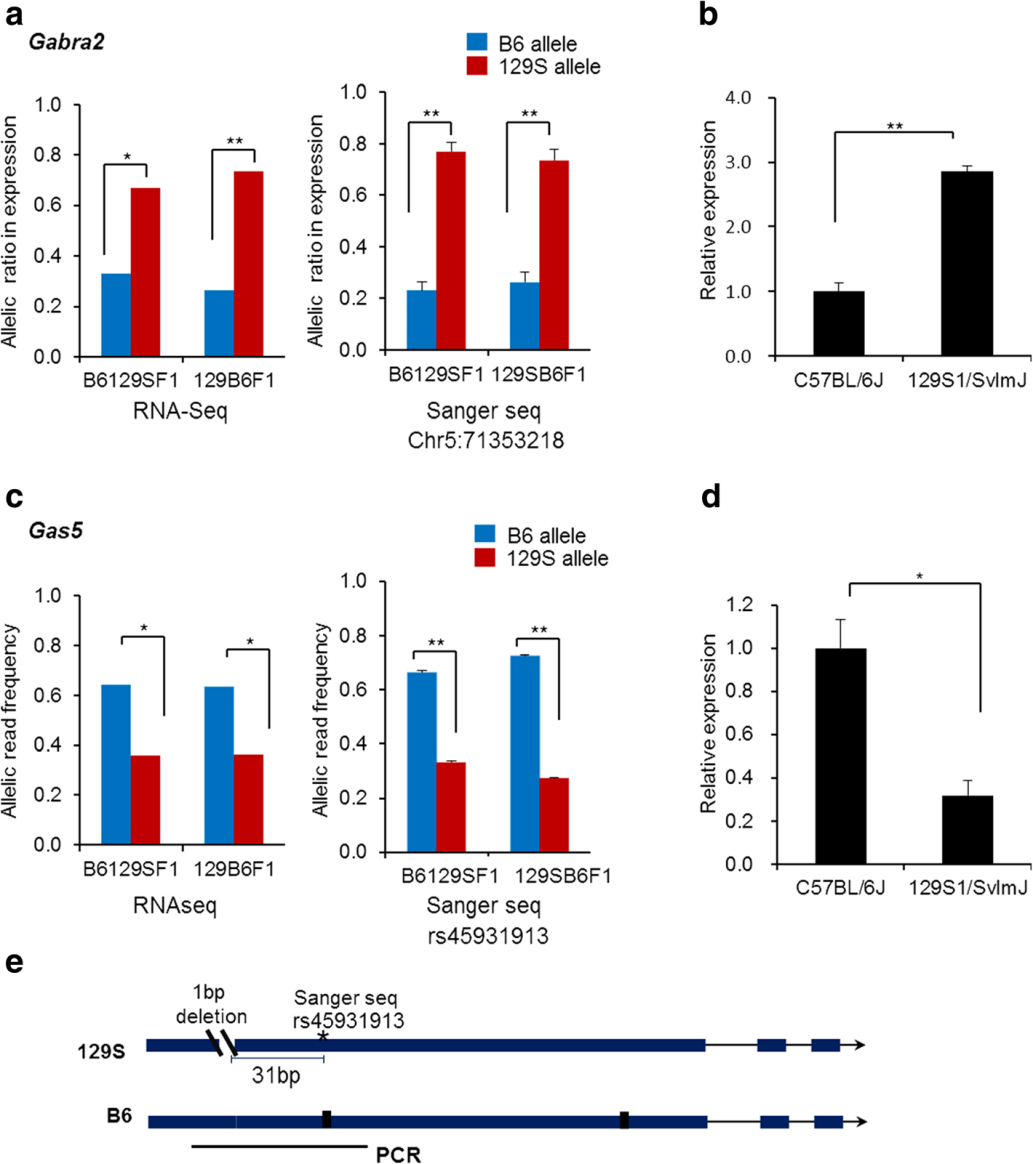
DAE QTLs and differential gene expression in the forebrain of B6/129SF1 mice and parental strains. **a** The 129S *Gabra2* allele was over-represented in reciprocal crosses when assayed either by RNA-Seq or by Sanger Sequencing. **b** qPCR in forebrain of the parental B6 and 129S strains shows that *Gabra2* is more highly expressed in the 129S strain. These data suggest the presence of a *cis*-acting regulatory element at *Gabra2* that is allelically variable between the two strains. **c** The B6 *Gas5* allele was over-represented in reciprocal crosses when assayed either by RNA-Seq or by Sanger Sequencing. **a**, **c** In RNA-Seq data, total numbers of reads of all SNVs in a gene were added up and then significance of DAE in the total number of reads was assessed by a binomial test. False Discovery Rate (FDR) adjusted p-values were obtained by the Benjamini-Hochberg method. The allele signals from the Sanger cDNA sequencing results were normalized using the allele-specific peak ratios from the Sanger gDNA sequencing. To test if the B6 allele and 129S allele were equally expressed, Sanger sequencing data were evaluated by a one-tailed *t*-test with values of B6 allele area/(B6 allele area + 129S allele area). **P < 0.005 and *P < 0.05 (**d**) qPCR analysis of forebrain of the parental B6 and 129S strains showed that *Gas5* is more highly expressed in the B6 strain. (B,D) Data of qPCR are the means ± SEM of 4 or 5 mice of each 129S1/SvlmJ and C57BL/6 J. **P < 0.005 and *P < 0.05 by a two-tailed *t*-test. These data suggest the presence of a *cis*-acting regulatory element at *Gas5* that is variable between the two strains, however (**e**) the presence of a 1 bp deletion in the 5’-UTR of 129S transcript may lead to transcript instability resulting in the observed allelic expression imbalance in F1’s and difference in expression between parental strains, as discussed

Several genes known to play critical roles in neural functions and behavior exhibited DAE QTLs in forebrain of B6/129SF1 mice. *Gabra2* encodes the gamma-aminobutyric acid (GABA) receptor α-2 subunit. GABA receptors are ligand-gated chloride ion channels that mediate GABA inhibitory neural transmission and that have been shown to influence alcohol and other addictive substance use. A negative correlation between oral morphine consumption and *Gabra2* expression was observed in inbred mouse strains [24, 25]. In B6/129S F1 mice, the 129S allele is expressed at levels 4-fold higher than the B6 allele in both reciprocal crosses (Fig. 3). This finding is consistent with the previously observed behavioral differences between the strains, and with the observation that *Gabra2* expression differs between C57BL/6J and 129S1/SvlmJ strains. This could be a *trans*-regulatory effect, but our data suggests that the observed strain-specific differences in *Gabra2* expression arise due to the presence of *cis*-acting variants at or near the *Gabra2* gene.

Variation at the *Gas5* gene (growth arrest specific 5) has been associated with levels of aggressive behavior in mice. This non-coding RNA was shown to be expressed at levels 8-fold higher in the brains of highly aggressive mouse strains than in less aggressive strains [26]. B6 mice have been reported to exhibit higher levels of aggressive behavior than 129S mice [27]. Our data showed both higher levels of *Gas5* expression in forebrain of B6 compared to 129S mice, and that the B6 allele is preferentially expressed in the forebrain of both reciprocal B6/129S F1 crosses (Fig. 3). This suggests that a *cis*-element in B6 allele promotes stronger transcription of the *Gas5* gene. However, an alternative explanation is that a deletion (chr1:162,966,110) in the *Gas5* gene of 129S mice may lower the stability of the 129S-derived transcript, which calls attention to the need to consider non-transcriptional mechanisms for eQTLs.

### Genetic association with phenotype (QTLs) in crosses between inbred strains of mice and DAE QTLs in F1 mice as evidences of QTLs

Genomic loci modulating several behavioral phenotypes have been mapped using genetic markers in crosses of inbred mouse strains and in recombinant inbred (RI) lines such as the BXD lines and more recently the Diversity Outbred (DO) crosses. Mapping these behavioral quantitative trait loci (QTLs) has identified genomic regions containing multiple candidate genes for a variety of behaviors, including ethanol-related phenotypes [28, 29], cocaine-related phenotypes [30], and seizure susceptibility [31]. A survey of the Mouse Genome Informatics (MGI) database identified 131 QTLs covering 45 different phenotypes (response to alcohol, morphine, cocaine, or methamphetamine, and other neurobehavioral phenotypes including seizures, sleep, and circadian rhythm). The QTL findings distinguishing B6 mice and DBA mice implicate some 415 candidate genes in crosses involving the B6 and DBA strains (Additional file 1: Table S5). Of these 415 candidate genes 91 were assayable for DAE in forebrain, using our stringent criteria, and 61 were assayable in kidney. The remainder either lacked the prerequisite genetic variation for assay, or was not expressed at a sufficient level. Four of the 91 candidate forebrain genes were DAE QTLs in the B6/DBA F1 mouse forebrain: *Darc* (duffy antigen receptor for chemokines), *Kifap3* (kinesin-associated protein 3), *Atp1b1* (sodium/potassium-transporting ATPase subunit beta-1 and *Ndn* (necdin), indicative of the presence of *cis*-acting regulatory alleles different the parental strains (Additional file 1: Table S6). *Atp1b1* showed elevated levels of the DBA derived transcript in both forebrain and kidney (Additional file 2: Figure S6). The fraction of QTL-implicated candidate genes exhibiting DAE was higher than would be expected by chance. In brain, 4.4 % (4/91) of the candidate genes were DAE QTLs as compared to 1.9 % (51/2669) overall (*p* = 0.0008). Similarly, in kidney, 21.3 % (13/61) of the QTL-implicated candidate gene genes were DAE QTLs versus 9.1 % (203/2236) overall (*p* = 0.019).

Several of the genes that we find differentially expressed in mouse forebrain have been previously implicated in alcohol preference via alcohol QTLs and shown to differ in expression in B6 mice as compared to other strains via conventional transcript measures. DAE in B6/DBAF1 mice confirms previous findings that *Darc*, *Kifap3*, *Aldh9a1*, *Sc5d*, *Sdhc* and *Sorl1* are transcribed at different levels in B6 and DBA brains between B6 and DBA mice [28, 29], a difference arising due to the presence of *cis*-acting loci at these genes (Additional file 2: Figure S6). 129S and DBA mice show lower alcohol preference compared to B6 mice [32–34], and we observe that *Darc*, *Sdhc*, and *Sc5d*, all candidate genes at alcohol related QTLs, have DAE QTLs in the forebrain and kidney of both B6/DBA and B6/129S F1 mice.

*Hdc* (histidine decarboxylase), a candidate gene within the *Pbrgcsf1* QTL (peripheral blood stem cell response to granulocyte colony stimulating factor 1) [35], has been shown to differ in expression between inbred mouse strains. Hdc protein levels and enzymatic activities of the DBA/2J mouse are approximately 10-fold higher than in C57BL/6J but only in the kidney [36]. Consistent with these findings, we observed that the DBA allele of *Hdc* is almost exclusively expressed in kidney of B6/DBAF1 mice (Additional file 2: Figure S6) suggesting the existence of a DBA specific, *cis*-acting regulatory sequence variant.

However, most assayable candidate genes within the identified QTLs for complex phenotypes did not show DAE QTLs in B6/DBAF1 mice (Additional file 1: Table S7) even though several of these genes previously were flagged as showing higher or lower transcript levels in C57BL/6J mice compared to DBA/2J mice [28]. This may suggest a difference in methodological precision of DAE versus measures of transcript level, or more intriguingly that the differences in transcript levels were caused by *trans*-acting factors, and even though the genes were located within behavioral QTLs. The fact that a gene exhibiting altered transcript levels is localized to a QTL does not necessarily mean that the sequence variant altering level of the transcript is local to the gene. For example, *Syn3* was identified as a candidate gene associated with a reversal-learning phenotype in the BXD RI strains based on its being one of five genes under a behavioral QTL peak, and because its level of expression correlated with the behavioral phenotype in BxD strains [37]. However, our data reveal no DAE for *Syn3* in the B6/DBA F1 mice (Additional file 2: Figure S7), indicating that at least for this candidate gene the difference in RNA expression which correlates with the phenotype may not be due to a *cis*-acting regulatory variant, but may arise through a *trans* mechanism or may have been an artifact chance finding. Most likely, many of the gene expression differences observed between strains and implicating genes that map to QTLs are not due to the actions of *cis*-acting loci at these genes. We note, however, that it is possible that an action of a *cis*-locus to drive DAE might be observed in other brain regions. DAE based on RNA-Seq of one brain region, or even several, cannot rule out the existence of *cis*-acting loci operative in some region of the brain or a particular cell. However, where a DAE QTL is observed, it strongly points to the existence of such a *cis*-locus. Although *cis*-effects will not be observed for most genes within QTL intervals, using RNA-Seq in F1 animals we were able to observe *cis*-effects for candidate genes related to alcohol, seizures, angiogenesis, and peripheral blood stem cell response (Additional file 1: Table S6).

### *Cis*-eQTLs in RI and F2 databases vs DAE QTLs in F1 mice

**Fig. 4:**
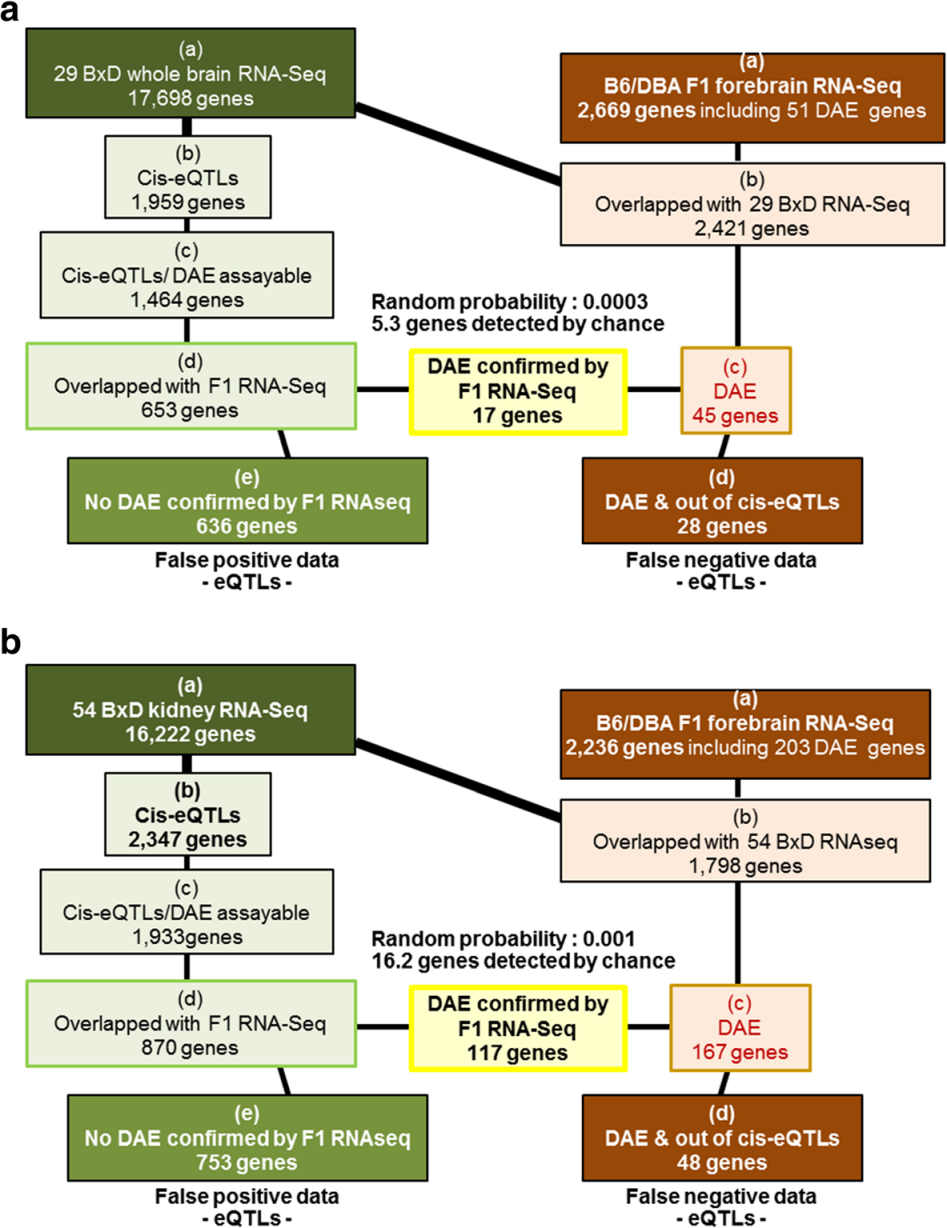
Comparison of DAE QTLs of B6/DBAF1 mice with autosomal *cis*-eQTLs of BxD RI mice. (**a**) forebrain and (**b**) kidney. BXD RNA-Seq data (*green boxes*) showing (a) total number of detected genes, (b) total number of genes, at *cis*-eQTLs, (c) number of DAE assayable genes at *cis*-eQTLs, (d) number of genes, at *cis*-eQTLs, overlapping with DAE QTLs in F1 RNA-Seq, (e) number of genes, at cis-eQTLs, which were not confirmed as DAE QTLs. Data from DAE QTLs detected by B6/DBAF1 RNA-Seq (brown boxes) showing (a) total number of detected genes, (b) number of genes detected in BXD and F1 RNA-Seq, (c) number of DAE QTLs, (d) number of DAE QTLs, which were not detected as *cis*-eQTLs. The yellow box shows the number of genes identified in both *cis*-eQTLs and DAE QTL analyses. The degree of overlap between the two approaches is significant in both forebrain (*p* = 0.0003) and kidney (*p* = 0.001)

Multiple expression quantitative trait loci (eQTLs) have been identified by correlating genotype data with gene expression in F2 crosses and RI strains. Using RNA-Seq DAE we estimated the number of genes with eQTLs in BXD RI strains that represent genuine *cis*-eQTLs using the BXD eQTLs listed in GeneNetwork (www.genenetwork.org). In GeneNetwork the BXD brain *cis*-eQTLs are based on data from 29 BXD RI strains, collected from three RNA-Seq datasets. The kidney *cis*-eQTLs in this database are derived from 54 BXD RI lines collected from three RNA micro array datasets. Out of the 26,378 autosomal genes listed in GeneNetwork, 17,698 and 16,222 were found as eQTLs in whole brain and kidney, respectively (Fig. 4), and of which 1,959 in whole brain and 2,347 in kidney are listed as *cis*-eQTLs. Only 1,464 genes in brain (74.7 %) and 1,933 in kidney (82.4 %) were potentially assayable by DAE, and of these only 653 genes in brain and 870 genes in kidney were available to observe in our B6/DBAF1 RNA-Seq. 45 of 51 DAE QTLs in forebrain and 167 of 203 DAE QTLs in kidney were listed as eQTLs. 17/653 assayable whole brain *cis*-eQTLs and 117/870 assayable kidney *cis*-eQTLs were confirmed by DAE QTLs. The rate of confirmation was above random expectations (brain: *p* = 0.0003, kidney: *p* = 0.001), the randomly expected overlap being only 5.3 and 16.2 genes in brain and kidney respectively, as compared to 17 and 117 genes observed. However, 97 % (636/653) of assayable forebrain *cis*-eQTLs and 87 % (753/870) of assayable kidney *cis*-eQTLs could not be confirmed by DAE. Overall, these data suggest that *cis*-eQTL mapping, using a restricted number of RI strains in which RNA levels are quantitated, has a high false-positive rate. Additionally, 28 genes in forebrain and 50 genes in kidney showing DAE QTLs in B6/DBAF1 mice had not been reported as *cis*-eQTLs in BxD RI strains, and 6 imprinted genes, *C230091D08Rik*, *D7Ertd715e*, *Peg10*, *Peg13*, *Ragrf1*, and *Zdbf2* were identified only as eQTLs but not as *cis*-eQTLs, which suggests that *cis*-eQTL mapping in restricted numbers of RI strains also has a lower sensitivity to detect *cis*-acting regulatory elements than DAE performed in a single reciprocal F1 cross. The discrepancy between reported *cis*-eQTLs and our DAE *cis*-eQTLs could arise from several confounding factors including lack of LD between cSNV reporters and regulatory loci in DAE analysis, inadequate sequencing in DAE coverage leading to failure to detect more subtle allelic differences, and as is likely important, false positive and false negative *cis*-eQTLs from studies of small numbers of RI strains.

## Discussion

Using RNA-Seq based transcriptome analysis, we performed genome-wide DAE to identify *cis*- regulated genes in the forebrain and the kidney of F1 mice generated through reciprocal crossing of C57BL/6J to one of three other inbred strains: 129S1/SvlmJ, DBA/2J and CAST/EiJ. 129S1/SvlmJ and DBA/2 J differ from C57BL/6J in several phenotypic traits including alcohol preference, anxiety behaviors, etc. [27, 33]. CAST/EiJ carries a greater number of genetic variants compared to the three inbred strains [38]. We used stringent filters to identify *cis*- regulated genes in the F1 mice to reduce the likelihood of false positive results, and even of the extreme of assayability. These filters included independent verification of SNVs from the Sanger database and exome sequencing of the parental inbred mice, cross- validation of allelic expression in the two reciprocal F1 strains, and use of an FDR threshold ≤ 0.05. These measures allowed us to identify genes with strain-specific and tissue-specific allelic expression and imprinted genes with high accuracy, and in strong correlation with differential gene expression in the parental strains.

The DAE QTLs we identified showed several validating features. Firstly, they could be verified by several factors such as consistent maternal-allelic expression of X-linked genes in F1 male mice (Additional file 2: Figure S3) and highly consistent correlation (*r* = 0.89, *p* = 5.4E-15) between Sanger sequencing and RNA-Seq. Moreover, we also observed that genes with strain-specific allelic expression in F1 mice were highly differentially expressed between the parental inbred strains with significant correlations between the qPCR results in the inbred strains and DAE results in F1 mice (*r* = 0.7, *P* = 0.014) (Additional file 2: Figure S4). Those results ensure that the observed DAE QTLs in F1 mice are represent authentic DAE and the action of *cis*-acting loci.

An important piece of evidence supporting the sensitivity and specificity of DAE QTLs was the successful detection of the imprinted genes exhibiting allele-specific transcription depending on maternal or paternal origin [39]. The number of imprinted genes expressed in brain remains uncertain, but our detection of 16 imprinted genes in B6/CASTF1 forebrain, is in line with a recent analysis [16], indicating that parentally imprinted genes number in the dozens, rather than hundreds, or even thousands reported elsewhere [13]. An important methodological caveat is that we analyzed expression in forebrain (as well as kidney), rather than in whole brain which is where the data reporting the much larger number of imprinted genes was derived. Of the 16 imprinted genes identified in our study, 13 were previously shown to be imprinted by DeVeale and colleagues (Additional file 1: Table S8). The smaller number of imprinted genes detected in our study reflects technical factors including sequencing coverage and the highly stringent filters used for SNV inclusion to reduce false positives, as also led to a lower number of DAE assayable genes. It should be noted that earlier studies also identified fewer imprinted genes in adult prefrontal cortex and preoptic area than were found in the developing E15 brain. Because only 4,171 genes were assayable for DAE in our study, the total number of imprinted genes may be higher, but is unlikely to number in the thousands. We also detected imprinting of 4 non-coding transcripts, suggesting that other noncoding transcripts not assayable for DAE in our study might also be imprinted. It has recently been reported that random mono allelic expression (RMAE) is widespread in lymphoblastoid cell lines derived from F1 mice generated by mating a129S1/SvlmJ dam to a Cast/EiJ sire, and that approximately 10 % of assessed genes showed DAE [40]. Surprisingly, although each clone of lymphoblastoid cell lines had the same genetic background, each clone had different patterns of differential allele expression across the same genes [40]. It has been hypothesized that various factors in cell culture conditions might drive this observed increase in variability in DAE [41, 42]. Despite the fact that forebrain consists of a mixture of different cell types (e.g., cholinergic, GABAergic neurons), we detected consistent patterns of DAE by RNA-Seq and Sanger sequencing using the forebrain of the same six F1 mice. This indicates that strain-specific DAE QTLs are consistent among F1 mice with the same genetic background.

Thirdly, we observed more tissue-specific DAE QTLs than non-tissue specific DAE QTLs (Fig. 2). This suggests that each tissue has tissue specific trans-factors influencing allelic expression, which shows B6 allele specific, alternative allele specific or bi-allelic manner in those F1 mice. These findings point to complexities of regulatory networks that affect tissue-specific expression at both gene and allelic levels.

Our strain-specific DAE QTLs showed correlations with strain-specific differences in gene expression that have previously been to be associated with strain-specific phenotypes. Genetic variations at *Gas5* and *Gabra2* are associated with the more aggressive behaviors of C57BL/6J and with the lower oral morphine consumption in 129S/SvlmJ compared to C57BL/6J, respectively [24–26]. Although the differences in behaviors between C57BL/6J and 129S1/SvlmJ can be correlated with differential gene expression between the strains [24–26], our F1 RNA-Seq data revealed that differential gene expression arose due to *cis*-acting genetic variants at those loci (Fig. 3).

Understanding the genetic basis of phenotypic variation is crucial to a number of questions in behavioral ecology. Many genes may influence a behavior, and among there it is important to identify both coding sequence and regulatory variants. Of the genes in phenotypic QTLs that were assayable by DAE, 4/91 in forebrain and 13/61 in kidney actually were DAE eQTLs, representing candidate *cis*-acting loci for the phenotypes. Failure to detect a DAE QTLs in F1 mice does not, however, mean that a *cis* eQTL is not present, for the reason of sequencing coverage or R^2^ to the DAE SNVs, or cell or tissue source of the RNA. Obviously, the molecular mechanism that gives rise to the behavioral QTL might not be related to RNA expression levels (Additional file 1: Table S5). However, our RNA-Seq DAE analysis did reveal that several candidate genes within QTLs associated with alcohol-drinking, seizure, granular brain lesions, peripheral blood stem cell response to G-CSF1 and kidney activity (Additional file 1: Table S6) were regulated by *cis*-acting loci. For example, *Darc*, *Sdhc*, and *Sc5d*, candidate genes, which lie within alcohol-drinking QTLs, consistently showed DAE in both B6/DBAF1 mice and B6/129SF1 mice. Because DBA/2J mice and 129S1/SvlmJ mice show lower alcohol preference compared to C57BL/6J mice, DAE of those candidate genes could potentially explain the phenotypic trait differences between the strains. The correlation between DAE in the F1 mice and behavioral differences in their parental strains not only validate our DAE findings, which were also consistent with the differential gene expression in the parental strains, but also suggest the value of genome-wide DAE to uncover genetic mechanisms modulating gene expression and heritable phenotypes.

DAE analysis may potentially provide more direct evidence of *cis*-regulation of genes than is provided by *cis*-eQTLs. DAE loci detected by RNA-Seq in B6/DBAF1 mice and *cis*-eQTLs (GeneNetwork) using BXD recombinant- inbred mice (RI) are generated with the similar aim of identifying of *cis*-acting functional loci. The practical consequence is that genetic and molecular methods can be used to cross-validate each other. Emphasizing the potential for the integration of the DAE QTLs with eQTLs from BXD recombinant-inbred mice (RI) (GeneNetwork), a comparison observed DAE loci in the B6/DBAF1 with BXD RI *cis*-eQTLs revealed significant overlap of findings in both forebrain (*P* = 0.0003) and kidney (*P* = 0.001) (Fig. 4), including several genes encoding GABA(A) receptor subunits. The B6 alleles of both *Gabrg2* and *Gabra1* were over-expressed compared to the DBA alleles [25, 43]. However, many assayable and previously listed *cis*-eQTLs could not be verified by DAE; 97 % (636/653 *cis*-eQTLs) in brain and 87 % (753/870 *cis*-eQTLs) in kidney. Focusing effort on genes that represent true-positives for functional *cis*-acting loci will hopefully lead to more rapid identification of causal sequence variants, and mechanisms by which these variants modulate phenotypes. It can also improve the power of gene network analysis, by identifying nodal genes whose differential expression is driven by *cis*-acting alleles, and that in *trans*-fashion may be driving expression of other genes within functional networks [44, 45]. In this regard, *cis*-eQTL mapping in RI strains did not detect certain genes with DAE QTLs, inclusion of which might strengthen gene network analyses. 28 DAE QTL genes in forebrain and 50 DAE QTL genes in kidney were not detected as *cis*-eQTLs even though these genes were annotated as eQTLs in the GeneNetwork eQTL dataset. Among the same categories of genes evaluated (assayable) by both methods, there are several potential explanations for mismatches between RI *cis*-eQTLs and DAE QTLs detected in F1 mice. These reasons include several methodological nuances in DAE: level of coverage and whether the reporter SNV is in high LD (r^2^) with the *cis*-locus altering expression. For *cis*-eQTLs derived from RI strains, a well-recognized limitation is restricted number of strains. Further, particular circumstances and criteria, by which RI eQTLs were generated, and complexity of genetic background (trans-effect) can mask *cis*-eQTLs.

RI strains have been an important resource for investigation and genetic mapping of Mendelian and quantitative traits in the mouse over the past several decades [46]. The RI panels provide numerous advantages, including the ability to study many animals of the same genotype. The additive genetic variance in a panel of RI strains is twice that of a corresponding F2 intercross for the same trait, a feature that increases the effective power to map subtle polygenic traits. Using RI strains, it is possible to reduce both environmental and technical sources of variance, leading to the detection of variants altering function, including gene expression [47]. However, the most noticeable disadvantage of RI strains has been the modest numbers of strains available per RI set, limiting their power to detect *cis*-eQTLs [48, 49]. Estimates of the effect size of QTLs identified in these RI panels are usually both noisy and upwardly biased (overestimated). One solution to this problem is to increase the size of RI strain panels [50]. However this requires significant resources and time for breeding and phenotyping. It does not eliminate the problem of trans-acting factors that may obscure *cis*-eQTLs or create false positives. The main advantages of DAE over eQTL mapping are the reduced number of animals required for the identification of *cis*-acting functional loci, and because the analyses are performed in F1 animals no recombination between chromosomes of the parental strains will have occurred, allowing the direct determination of the presence of *cis*-acting regulatory elements. DAE analysis in F1 inbred mice relies on the distribution of ratios of allele specific expression within each individual. DAE in F1 animals is readily adaptable to crosses made between RI strains, or between the parental strains, enabling the allele-specific effects on expression found by DAE in F1’s to be integrated with RI-based QTLs for behavior and other complex phenotypes.

Although DAE is an important addition to techniques for the identification of *cis*-acting regulatory elements, several limitations make it a technique that is complimentary to existing approaches such as eQTL mapping, rather than a universal method for identifying *cis*-acting alleles or imprinting. The primary limitation of DAE is that it requires the presence of at least one informative transcribed SNP. In outbred populations and for studies in humans this will often not be a problem. Additionally, it is also helpful, but not absolutely required for the reporter locus in strong LD with the functional *cis*-acting element. In order to take full advantage of DAE in human samples it is important that phase is known for all variants within a region. Unlike eQTL mapping, DAE directly identifies the presence of a *cis*-acting regulatory element, but like eQTL mapping, DAE does not directly identify the elements themselves that are acting to alter gene expression.

When adequately controlled for mapping bias, and other technical problems that limit assayability, DAE in F1 mice shows a high rate of specificity for detecting *cis*-acting loci, and the precision of the method, based as it is on transcript fragment counting, appears high. Our study using three different reciprocal F1 crosses shows that DAE is consistently observed in the same genomic background regardless of parental origin, unless there is imprinting. Intriguingly, pointing to cell-specific action, most DAE is not conserved between forebrain, an ectodermal derived tissue, and kidney, mesodermal derived tissue. Further study is required to truly understand how DAE QTLs in brain may be linked to behavioral phenotypes, including alcohol and drug responses and levels of anxiety that have been shown to vary among the inbred mouse strains we studied.

## Conclusions

Inbred mouse strains display differences in behavior, drug preference, and responses to alcohol. These strain differences are largely attributable to the genetic differences between them, including *cis*-acting elements that alter gene expression. Previous studies reported genetic variants associated with various phenotypes and differential gene expression through behavioral QTLs and *cis*-eQTLs, the validity and molecular mechanism remain further identification. Here, taking advantage of strain-specific genetic variation, we applied RNA-Seq in the reciprocally crossed F1 mice, to measure relative expression of alleles at heterozygous loci for differential allele expression (DAE) indicative of strain specific *cis*-acting loci altering RNA expression. We found that globally about 2 % of genes assayed in forebrain were regulated by *cis*-acting loci and most of the DAE genes showed tissue specificity. Also, through comparing our data with previous phenotype QTLs and *cis*-eQTLs, we have built on the literature showing DAE analysis is valuable in identifying and confirming *cis*-acting regulated genes that have been associated with specific phenotypes shown by the classic QTL analysis.

## Methods

### RNA-Seq library construction

All research protocols involving live animals were approved by the institutional review board of National Institute on Alcohol Abuse and Alcoholism. F1 mice were generated by reciprocal crosses between C57BL/6J and three other strains of inbred mice, 129S1/SvlmJ, DBA/2J and CAST/EiJ (Jackson Laboratories). Eight week old F1 mice, including 6 male mice from each reciprocal cross, were sacrificed by cervical dislocation. Forebrain and kidney were dissected and total RNA extracted using the mirVana miRNA isolation kit (Ambion), according to the manufacturer’s protocol. Aliquots of total RNA from all 6 animals for each F1 cross were pooled. To eliminate residual genomic DNA, RNA samples were incubated with DNase (Ambion) at 37 °C for 30 min. mRNA was isolated from 20 μg of total RNA using MicroPoly(A) Purist kit (Ambion, City, USA) and purified with Ribominus concentration module (Invitrogen). The purified mRNA was fragmented to 100 ~ 200 bp in length, and adaptors (Illumina) were ligated before reverse transcription to generate libraries according to the protocol of Ambion RNA-Seq Library construction. cDNA fragments of 200–300 bp were isolated using Novex® 6 % TBE-Urea Gel. The libraries were amplified by PCR for 16 cycles, quantified by Qubit dsDNA BR (Invitrogen), and profiled by Bioanalyzer DNA 1000 kit (Agilent).

### Exome-seq libraries

Genomic DNA (gDNA) was extracted from spleen of 8 week old mice representing each parental inbred strain (DBA/2J, CAST/EiJ, 129S1/SvlmJ and C57BL/6J male mice). 3 μg of gDNA was sheared with Covaris S-series single tube sample preparation system (Covaris) to 150 bp fragments. Fragmented gDNA was used to make Exome libraries using SureSelect XT Mouse All Exon kit (Agilent Technologies) according to the manufacturer’s protocol. Briefly, DNA was end-repaired and phosphorylated. After A-tailing, genomic DNA fragments were ligated to sequencing adapters. Ligated genomic DNA fragments were amplified by PCR for 5 cycles using Herculase II Fusion DNA Polymerase (Agilent). DNA libraries were purified with Agencourt® XP® Beads (Beckman Coulter Genomics) after each enzymatic reaction and the amounts and sizes of PCR products were measured using an Agilent 2100 Bioanalyzer (Agilent Technologies) and Qubit assays (Life Technology). Exonic regions were captured by hybridizing 500 ng of genomic DNA libraries with a Sure Select mouse All Exon bait library (Agilent). Captured libraries were amplified for 11 cycles by PCR. The exome libraries were analyzed for fragment sizes and concentrations using an Agilent Bioanalyzer High Sensitivity DNA kit (Agilent Technologies) before sequencing.

### RNA-Seq and exome-Seq: sequencing and data processing

5 ng of the RNA-Seq libraries and Exome-Seq libraries were used for cluster generation on a grafted GAII Flow Cell, and then sequenced for 36 cycles on the Illumina GAIIx Genome Analyzer. Sequence was called with CASAVA v1.8.1 (Illumina), and mapped to the B6 mouse reference sequence (UCSC mm9) and the alternative reference based on 129S1/SvlmJ, DBA/2J, and CAST/EiJ genome sequences (Sanger Institute, REL-1105). SNVs were identified using CASAVA v1.8.1 with variants NoCovCutoff option. A total of 125,640 single nucleotide variants (SNV) were identified with minimum coverage ≥4 and Q (snp) ≥20. SNVs near splicing sites in introns from RNA-Seq reads were excluded.

To minimize bias caused by differences in genetic distance between the parental genomes and the reference sequence and to improve alignment quality, alternative references were constructed by substituting known strain specific SNVs (Sanger Mouse Genomes Project, REL-1105). Reads of each F1 RNA-Seq were aligned to the both B6 reference (mm9) and the alternative reference for the non-B6 parental inbred strain of the F1 mice. The average number of mapped reads from two references was used as the mapped reads at each SNV. Finally, we selected SNVs that were confirmed by Exome-Seq or/and Sanger mouse SNV database and had number of reads ≥8. Total numbers of reads of all SNVs in a gene were added up and then significance of DAE at each gene was assessed by a binomial test. In addition, False Discovery Rate (FDR) adjusted p-values were obtained by the Benjamini-Hochberg method [51].

### Quantitative Sanger sequencing to detect allelic imbalance in cDNA

Genomic DNA, to be used as a control, was extracted from spleen of six 129B6F1 and six B6129SF1 male mice using the SureSelect gDNA Extraction Kit (Agilent Technologies). Total RNA was extracted from the forebrain from six 129SB6F1 and six B6129SF1, which were the same mice for RNA-Seq, using mirVana miRNA isolation kit (Ambion). cDNA was synthesized by reverse transcription of 2 μg of total RNA extracted from the forebrain of the same mice with High-capacity cDNA Reverse Transcription kits (Applied Biosystems). PCR amplification was performed using AmpliTaq Gold DNA polymerase (Applied Biosystems), 10 ng of template, 1 μl of forward and reverse primer (5 μM each), 2 μl of H_2_O at 94 °C for 1 min, followed by 40 cycles each at 94 °C for 20 s, 60 °C for 30 s, 68 °C for 30 s, and a final extension step of 72 °C for 10 min. Primer sequences are listed in Additional file 1: Table S9. The PCR products (200 ~ 350 bp) were purified using the USB ExoSAP-IT reagent (Affymetrix) according to the protocol and sequenced on an Applied Biosystems 3730 DNA Analyzer with Big Dye Terminator Sequencing Mix (Applied Biosystems). In order to measure DAE in Sanger sequencing electropherograms, areas under the peaks at target positions were measured using ImageJ1.46r (Wayne Rasband, National Institutes of Health, http://imagej.nih.gov/ij/). The allele signals from the Sanger cDNA sequencing results were normalized using the allele-specific peak ratios from the Sanger gDNA sequencing. To test if the B6 allele and 129S allele were equally expressed, Sanger sequencing data were evaluated by a one-group *t*-test with values of B6 allele area/(B6 allele area + 129S allele area). The analyses were undertaken using JMP11 software (SAS Institute).

### Quantitative RT-PCR

As biological replicates, 4 or 5 mice representing each strain (129S1/SvlmJ and C57BL/6J) (8 week old) were used to extract total RNA of forebrain with the same method as described above. Residual genomic DNA contamination was removed by incubation of RNA samples with DNaseI (Ambion) at 37 °C for 30 min. 2 ug of total RNA was reverse-transcribed into first strand cDNA with the High Capacity cDNA reverse transcription kit (Applied Biosystems) at 25 °C for 10 min, 37 °C for 2 h, and 85 °C for 5 min, in a 20 μl reaction volume. For quantitative PCR, 1 μl of cDNA was mixed with gene-specific primers (0.25 μM final concentration), 2x SYBR Green master mix (Applied Biosystems) in a 10 μl volume. Reactions were carried out on the ABI 7900 real-time PCR system with the following protocol: one cycle of 95 °C for 3 min, 40 cycles of 95 °C for 15 s and 60 °C for 1 min. Primer sequences are listed in Additional file 1: Table S9. Relative gene expression levels for each sample were calculated using the ∆Ct values (normalized detection threshold). After normalization with expression of beta-actin, each value of C57BL/6J and 129S1/SvlmJ was re-normalized based on the level of gene expression in C57BL/6J. Quantitative RT-PCR data were assessed by a two-group *t*-test (Additional file 1: Table S10).

### Behavior and other phenotype-related QTL data

Except for eQTLs, mouse phenotype QTLs, previously detected in BXD mice or F2 mice generated between C57BL/6J and DBA/2J, were extracted from the Mouse Genome Informatics (MGI) database (www.informatics.jax.org/allele/) [29]. We selected QTLs reported with candidate genes, because some QTLs were not accompanied by a reported candidate gene. We evaluated the candidate genes for these phenotypes against the DAE QTLs we detected in B6/DBAF1 mice. To compare eQTL data with DAE QTLs, we used BxD brain *cis*-eQTLs and kidney *cis*-eQTLs from ‘QTL miner’ in GeneNetwork (www.genenetwork.org). BxD brain *cis*-eQTLs in GeneNetwork are based on three RNA-Seq datasets (UTHC mouse BXD whole brain RNA-Seq (Nov12) RPKM Untrimmed, UTHC mouse BXD whole brain RNA-Seq (Nov12) RPKM Trimmed, and UTHC mouse BXD whole brain RNA-Seq exon level (Nov12)RPKM with 29 BXD RI (recombinant inbred) mice. BXD kidney *cis*-eQTLs in GeneNetwork are derived from three RNA micro array datasets (Mouse Kidney M430V2 Male (Aug06) RMA, Mouse Kidney M430V2 Sex Balanced (Aug06) RMA, and Mouse Kidney M430v2 (Jul06) RMA) with 54 BXD RI (recombinant inbred) mice. From the expressed genes in brain or kidney, we collected genes marked in GeneNetwork as ‘*cis*-regulated’ and compared each gene symbol with our B6/DBAF1 DAE QTLs.

## Abbreviations

DAE, differential allelic expression; eQTL, expression quantitative trait loci; QTL, quantitative trait loci; RI, recombinant inbred; SNP, single nucleotide polymorphism; SNV, single nucleotide variant

## Additional files

Additional file 1: Table S1. RNA-Seq depth in forebrain and kidney of reciprocal F1 crosses. **Tabl S2.** SNVs detected by exome sequencing (Exome) or/and Sanger mouse SNVs database (Sanger) in three inbred mouse strains. **Table S3.** List of imprinted genes containing SNVs, which were observed with 8 reads in forebrain RNAseq. **Table S4.** Statistically significant evidence of DAE ratios diverging from 1:1 observed in reciprocal crosses. **Table S5.** Phenotype-related QTLs reported in BxD RI mice or F2 mice derived from B6 and DBA. **Table S6.** Genes at phenotype QTLs have DAE QTLs. **Table S7.** Genes implicated as phenotypic QTLs showing no DAE in (A) forebrain and (B) kidney of B6/DBA2 F1 mice. **Table S8.** 16 Imprinted genes (including four imprinted non-coding RNAs) detected by brain RNA-Seq of B6/CASTF1. **Table S9.** Primers for differentially allelic expression analysis or quantitative PCR. **Table S10.** Quantitative PCR verification of 10 DAE QTLs in parental strains, C57BL/6J and 129S1/SvlmJ. (ZIP 639 kb) Additional file 2: Figure S1. Correction of mapping bias in RNA-Seq using strain specific genomic information. **Figure S2.** Volcano plots identifying DAE loci. **Figure S3.** X-linked genes expressed in forebrain and kidney show uni-parental (maternal) expression in male F1 mice. **Figure S4.** Validation of 20 DAE QTLs and 15 non-DAE genes in forebrain of B6/129SF1 mice. **Figure S5.** Validation of DAE for imprinted genes in the forebrain of B6/129SF1 mice. **Figure S6.** Candidate genes at QTLs show DAE in both forebrain and kidney. **Figure S7.** *Syn3*, a gene located at a QTL associated with learning behavior shows no allelic imbalance in forebrain or kidney. Figure S3 *Zim3* is expressed from only the 129S chromosome in forebrain of reciprocal F1 mice, potentially due to an INDEL in 129S gDNA. (ZIP 1175 kb)
