# Supplementary material for: The abundance of cis-acting loci leading to differential allele expression in F1 mice and their relationship to loci harboring genes affecting complex traits
Source: BMC Genomics. 2016 Aug 11;17:620. doi: 10.1186/s12864-016-2922-9 (PMC4982227; doi:10.1186/s12864-016-2922-9)
Supplement: Additional file 1: Table S1. — RNA-Seq depth in forebrain and kidney of reciprocal F1 crosses. Tabl S2. SNVs detected by exome sequencing (Exome) or/and Sanger mouse SNVs database (Sanger) in three inbred mouse strains. Table S3. List of imprinted genes containing SNVs, which were observed with 8 reads in forebrain RNAseq. Table S4. Statistically significant evidence of DAE ratios diverging from 1:1 observed in reciprocal crosses. Table S5. Phenotype-related QTLs reported in BxD RI mice or F2 mice derived from B6 and DBA. Table S6. Genes at phenotype QTLs have DAE QTLs. Table S7. Genes implicated as phenotypic QTLs showing no DAE in (A) forebrain and (B) kidney of B6/DBA2 F1 mice. Table S8. 16 Imprinted genes (including four imprinted non-coding RNAs) detected by brain RNA-Seq of B6/CASTF1. Table S9. Primers for differentially allelic expression analysis or quantitative PCR. Table S10. Quantitative PCR verification of 10 DAE QTLs in parental strains, C57BL/6J and 129S1/SvlmJ. (ZIP 639 kb) [file 12864_2016_2922_MOESM1_ESM.zip › add2/Additional file 4_Figure S4.pdf]

A

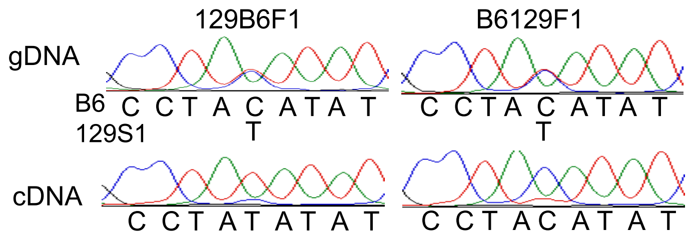

Measurement (area under curve)	Deviation in measurement (with genomic DNA)	Corrected value (for cDNA)
Allele 1, a	a' = (c - a)/c	A = a + (a x a')
Allele 2, b	b' = (c - b)/c	B = b + (b x b')
	c = (a + b)/2	

B

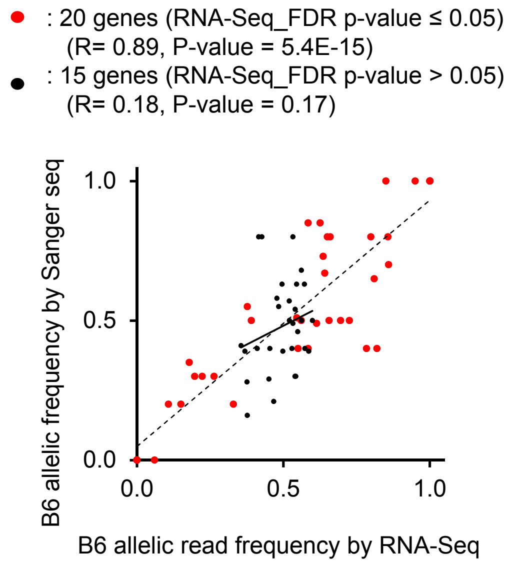

C

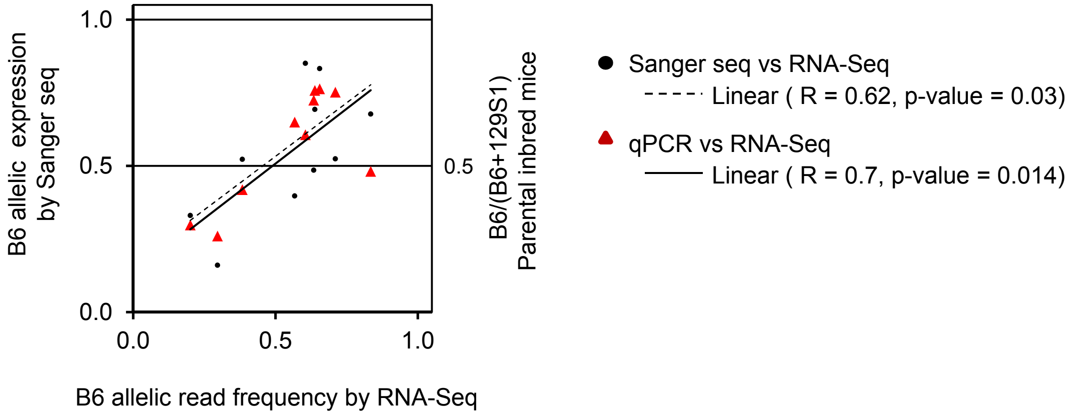

Figure S4. Validation of twenty DAE QTLs and 15 non-DAE genes in forebrain of B6/129SF1 mice. (A) Schematic diagram depicting how to analyze DAE by Sanger sequencing results (B) Validation of 20 DAE QTL genes and 15 genes not displaying DAE by Sanger sequencing. cDNA obtained from RNA of forebrain of B6129SF1 (n=6) and 129SB6F1 (n=6) mice, and genomic DNA derived from spleen of the same F1 mice was sequenced. The difference in gDNA allele-specific peak heights was used to correct bias in allele quantitation using the equation shown. Plotting B6 allele expression detected by fragment counting in RNA-Seq against the frequency determined by quantitative Sanger sequencing revealed strong correlation between Sanger quantitation and RNA-Seq quantitation of allelic imbalance, as shown. (C) Ten DAE QTLs showed differential gene expression between parental inbred mice (C57BL/6J_n=4 or 5, 129S1/SvlmJ_n=4 or 5). Correlation between RNA-Seq and Sanger-Seq (R=0.62, P-value = 0.03) for DAE genes (Black) and between RNA-Seq and relative expression in parental strains determined by qPCR (Red) (R=0.7, P-value = 0.014). Expression levels in parental strains were normalized to internal beta-actin controls and then expression values in 129S1/SvlmJ were normalized to the expression values in C57BL/6J, and the fractional expression of the B6 allele (B6/(B6+129S)) was calculated.
