# Supplementary material for: The abundance of cis-acting loci leading to differential allele expression in F1 mice and their relationship to loci harboring genes affecting complex traits
Source: BMC Genomics. 2016 Aug 11;17:620. doi: 10.1186/s12864-016-2922-9 (PMC4982227; doi:10.1186/s12864-016-2922-9)
Supplement: Additional file 1: Table S1. — RNA-Seq depth in forebrain and kidney of reciprocal F1 crosses. Tabl S2. SNVs detected by exome sequencing (Exome) or/and Sanger mouse SNVs database (Sanger) in three inbred mouse strains. Table S3. List of imprinted genes containing SNVs, which were observed with 8 reads in forebrain RNAseq. Table S4. Statistically significant evidence of DAE ratios diverging from 1:1 observed in reciprocal crosses. Table S5. Phenotype-related QTLs reported in BxD RI mice or F2 mice derived from B6 and DBA. Table S6. Genes at phenotype QTLs have DAE QTLs. Table S7. Genes implicated as phenotypic QTLs showing no DAE in (A) forebrain and (B) kidney of B6/DBA2 F1 mice. Table S8. 16 Imprinted genes (including four imprinted non-coding RNAs) detected by brain RNA-Seq of B6/CASTF1. Table S9. Primers for differentially allelic expression analysis or quantitative PCR. Table S10. Quantitative PCR verification of 10 DAE QTLs in parental strains, C57BL/6J and 129S1/SvlmJ. (ZIP 639 kb) [file 12864_2016_2922_MOESM1_ESM.zip › add2/Additional file 5_Figure S5.pdf]

**A**

| Gene | B6129SF1 | | | | 129SB6F1 | | | |
|---|---|---|---|---|---|---|---|---|
| | **Reads** | **B6 allele (maternal)** | **129S1 allele (paternal)** | **-logP*** | **Reads** | **B6 allele (paternal)** | **129S1 allele (maternal)** | **-logP*** |
| *Inpp5f* | 291 | 0.15 | 0.85 | 31.4 | 265 | 0.85 | 0.15 | 28.5 |
| *Zdbf2* | 31 | 0 | 1.00 | 7.1 | 28 | 1.00 | 0 | 6.3 |
| *Rasgrf1* | 320 | 0.06 | 0.94 | 62.9 | 245 | 0.95 | 0.05 | 49.5 |
| *Snrpn* | 44 | 0 | 1.00 | 10.7 | 27 | 1.00 | 0 | 6 |
| *Peg10* | 39 | 0 | 1.00 | 9.3 | 43 | 1.00 | 0 | 10.4 |
| *Sgce* | 17 | 0 | 1.00 | 3.3 | 12 | 1.00 | 0 | 2 |
| *C230091D08Rik* | 70 | 0.86 | 0.14 | 7.1 | 65 | 0.11 | 0.89 | 8.3 |
| *H13* | 124 | 0.80 | 0.20 | 8.8 | 132 | 0.20 | 0.80 | 9.8 |

*P-value: FDR adjusted p-value obtained by Benjamini-Hochberg method

**B**

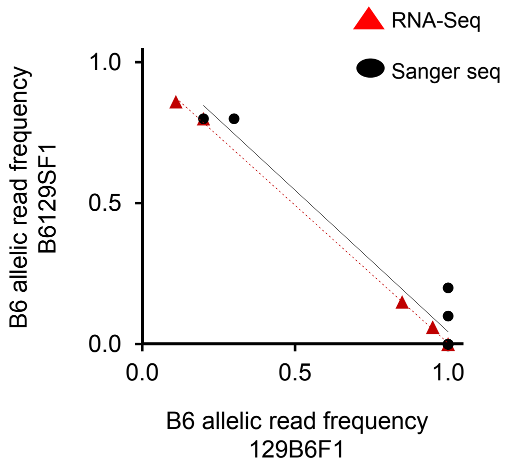


**Figure S5. Validation of DAE for imprinted genes in the forebrain of B6/129SF1 mice** (A) Eight genes detected by RNA-Seq to have parental imprinting in forebrain of B6/129S F1 mice, including 6 paternally imprinted and 2 maternally imprinted genes. (B) In each instance, the parentally-determined allelic imbalance in expression was replicated by quantitative Sanger Sequencing.
